# Correction: Kargul et al. Beyond Diameter: Enhancing Abdominal Aortic Aneurysm Surveillance with Volumetric Assessments After Endovascular Aneurysm Repair (EVAR). *J. Clin. Med.* 2023, *12*, 6733

**DOI:** 10.3390/jcm15156018

**Published:** 2026-08-03

**Authors:** Michał Kargul, Patryk Skórka, Piotr Gutowski, Arkadiusz Kazimierczak, Ireneusz Wiernicki, Paweł Rynio

**Affiliations:** Department of Vascular Surgery, Pomeranian Medical University in Szczecin, Al. Powstańców Wielkopolskich 72, 70-111 Szczecin, Poland

**Missing Acknowledgments**

In the original publication [1], the Acknowledgments was not included.

**Acknowledgments:** We would like to express our gratitude to Natalia Niklas-Maxia for her data collection efforts.

The authors state that the scientific conclusions are unaffected. This correction was approved by the Academic Editor. The original publication has also been updated.
